# Mitochondrial Ca^2+^ Signaling at the Tripartite Synapse: A Unifying Framework for Glutamate Homeostasis, Metabolic Coupling, and Network Vulnerability

**DOI:** 10.3390/biom16010171

**Published:** 2026-01-20

**Authors:** Mariagrazia Mancuso, Federico Mezzalira, Beatrice Vignoli, Elisa Greotti

**Affiliations:** 1 Neuroscience Institute, National Research Council of Italy, 35131 Padua, Italy; mariagraziamancuso@cnr.it (M.M.); federico.mezzalira@studenti.unipd.it (F.M.); 2 Department of Biomedical Sciences, University of Padova, 35131 Padua, Italy; 3 Padova Neuroscience Center, University of Padova, 35131 Padua, Italy

**Keywords:** astrocyte–neuron communication, glutamatergic synapse, mitochondrial signaling, glutamate homeostasis, metabolic coupling, mitochondrial Ca^2+^ signaling, excitotoxicity, neuronal hyperexcitability, synaptic vulnerability, Alzheimer’s disease

## Abstract

Mitochondrial Ca^2+^ signaling is increasingly recognized as a key integrator of synaptic activity, metabolism, and redox balance within the tripartite synapse. At excitatory synapses, Ca^2+^ influx through ionotropic glutamate receptors and voltage-gated channels is sensed and transduced by strategically positioned mitochondria, whose Ca^2+^ uptake and release tune tricarboxylic acid cycle activity, adenosine triphosphate synthesis, and reactive oxygen species (ROS) generation. Through these Ca^2+^-dependent processes, mitochondria are proposed to help set the threshold at which glutamatergic activity supports synaptic plasticity and homeostasis or, instead, drives hyperexcitability and excitotoxic stress. Here, we synthesize how mitochondrial Ca^2+^ dynamics in presynaptic terminals, postsynaptic spines, and perisynaptic astrocytic processes regulate glutamate uptake, recycling, and release, and how subtle impairments in these pathways may prime synapses for failure well before overt energetic collapse. We further examine the reciprocal interplay between Ca^2+^-dependent metabolic adaptations and glutamate homeostasis, the crosstalk between mitochondrial Ca^2+^ and ROS signals, and the distinct vulnerabilities of neuronal and astrocytic mitochondria. Finally, we discuss how disruption of this Ca^2+^-centered mitochondria–glutamatergic axis contributes to synaptic dysfunction and circuit vulnerability in neurodegenerative diseases, with a particular focus on Alzheimer’s disease.

## 1. Introduction

Glutamate is the predominant excitatory neurotransmitter in the mammalian central nervous system and supports most information processing, from rapid sensorimotor responses to higher cognitive operations [1]. However, the very features that make glutamatergic signaling fast and flexible also render it intrinsically hazardous: because glutamate is both indispensable and potentially neurotoxic, its extracellular concentration must be tightly controlled. Even relatively small deviations can disrupt synaptic integration, alter circuit excitability, and initiate excitotoxic cascades: Ca^2+^-dependent degenerative processes that progressively erode neuronal circuits. This intrinsic duality—*essential yet dangerous*—has driven the evolution of a highly coordinated system of metabolic and signaling safeguards distributed across neurons, astrocytes, and mitochondria [2].

The tripartite synapse framework reconceptualized glutamatergic transmission as a coordinated interaction among presynaptic terminals, postsynaptic neurons, and perisynaptic astrocytic processes (PAPs) [3,4]. Astrocytes, once considered primarily supportive, are now recognized as essential regulators of glutamate homeostasis and active modulators of synaptic transmission via gliotransmitter release. Through the high-affinity excitatory amino acid transporters (EAATs)—EAAT1 (also known as glutamate-aspartate transporter, GLAST) and EAAT2 (also known as glutamate transporter-1, GLT-1)—they clear the majority of synaptic glutamate [1,5], thereby terminating synaptic transmission within milliseconds and preventing spillover, which would otherwise activate extrasynaptic N-methyl-D-aspartate receptors (eNMDARs) and downstream pro-death pathways [6]. Because each glutamate molecule is co-transported with Na^+^, uptake stimulates Na^+^/K^+^-ATPase activity and imposes one of the largest ATP (adenosine triphosphate) demands in the brain [7,8]. Internalized glutamate is converted to glutamine by glutamine synthetase (GS) and returned to neurons to sustain vesicle filling, closing the glutamate–glutamine cycle [9,10,11].

Neurons complement this architecture with a largely autonomous “glutamate economy”. Because circulating glutamate does not enter the brain [12], neurons synthesize transmitters either from tricarboxylic acid (TCA) cycle intermediates or from astrocyte-derived glutamine [1,10,11]. Synaptic vesicles are loaded via vesicular glutamate transporters (VGLUTs) using a proton gradient generated by the vesicular H^+^-ATPase, an energetically demanding process that depends on mitochondrial ATP supply [11,13,14,15,16,17,18]. Action potential-evoked Ca^2+^ influx through voltage-gated Ca^2+^ channels (VGCCs) creates Ca^2+^ nanodomains that activate the SNARE (soluble N-ethylmaleimide-sensitive factor attachment protein receptor) fusion machinery and trigger glutamate release with millisecond precision [19,20,21,22,23]. Vesicle retrieval, reacidification, and refilling impose further energetic demands that rely on both local glycolytic ATP and mitochondrial oxidative phosphorylation (OXPHOS) [24,25].

Postsynaptic neurons decode glutamate through ionotropic and metabotropic receptors. Ionotropic AMPA (α-amino-3-hydroxy-5-methyl-4-isoxazolepropionic acid), NMDA (N-methyl-D-aspartate), and kainate receptors (AMPARs, NMDARs, and KARs) mediate fast excitatory transmission. AMPARs and KARs generate rapid depolarization, whereas NMDARs uniquely couple glutamate binding to voltage-dependent Mg^2+^ relief and Ca^2+^ influx, thereby linking synaptic activity to intracellular signaling cascades underlying synaptic plasticity [26,27]. NMDAR-dependent long-term potentiation (LTP) is a long-lasting increase in synaptic strength induced by brief high-frequency or burst stimulation and provides a classical model of learning- and memory-related circuit modification [28,29]. Conversely, NMDAR-dependent long-term depression (LTD) reflects a persistent decrease in synaptic efficacy typically elicited by low-frequency stimulation and contributes to synapse weakening and circuit refinement [30].

Metabotropic glutamate receptors (mGluRs) provide a slower modulatory layer. Group I mGluRs (mGluR1/5) engage PLC (phospholipase C)-, MAPK (mitogen-activated protein kinase)/ERK (extracellular signal-regulated kinase)- and mTOR (mammalian target of rapamycin)-dependent pathways, whereas Group II/III mGluRs act predominantly as presynaptic autoreceptors to restrain glutamate release [31,32,33].

These processes operate at different temporal scales: glutamate release and EAAT-mediated clearance occur within milliseconds, whereas glutamate recycling and TCA cycle integration unfold over seconds to minutes [34,35]. This temporal mismatch makes excitatory signaling highly efficient but also intrinsically fragile, requiring rapid metabolic adjustments to maintain ionic and neurotransmitter homeostasis. Astrocytes partially address this imbalance through a fast metabolic response: glutamate uptake activates the Na^+^/K^+^-ATPase, accelerating glycolysis and lactate export to neurons [36]. However, the capacity of this compensatory response depends critically on the intact mitochondrial function.

Mitochondria, therefore, emerge as central integrators of glutamate homeostasis. They provide ATP for vesicle cycling, ion pumping, and EAAT-mediated uptake; buffer Ca^2+^ nanodomains to tune presynaptic release, postsynaptic plasticity, and gliotransmission; maintain redox balance; and metabolize glutamate via anaplerotic pathways that replenish TCA cycle intermediates [8,18,25,37]. Their strategic localization within boutons, spines and PAP-associated domains creates metabolic nanodomains capable of translating fast glutamatergic and Ca^2+^ signals into appropriately scaled metabolic responses, dynamically adjusting ATP production, NADH/NAD^+^ ratios, and reactive oxygen species (ROS) tone [18,38,39]. While these core functions are well supported experimentally, the precise spatial organization and compartment-specific integration of mitochondrial Ca^2+^, metabolic and redox signaling—particularly at fine perisynaptic scales—remain active areas of investigation.

Disruption of this astrocyte–neuron–mitochondria partnership is proposed to destabilize glutamate homeostasis, promote hyperexcitability, and lower the threshold for excitotoxicity. Importantly, physiological or compensatory hyperexcitability and excitotoxic degeneration are best viewed as points along a continuum: as long as mitochondrial and astrocytic safeguards can match glutamatergic load, increased excitability may remain reversible, whereas failure of these systems is thought to permit Ca^2+^ dysregulation, energetic collapse, and progressive synaptic damage to emerge. Emerging evidence suggests that such vulnerabilities are shared across multiple neurological conditions, with Alzheimer’s disease (AD) providing a paradigmatic example in which soluble amyloid-β (Aβ) oligomers, Tau pathology, apolipoprotein E (APOE) ε4 and aging-associated metabolic decline converge to erode glutamate clearance and mitochondrial resilience [40,41,42].

**Figure 1:**
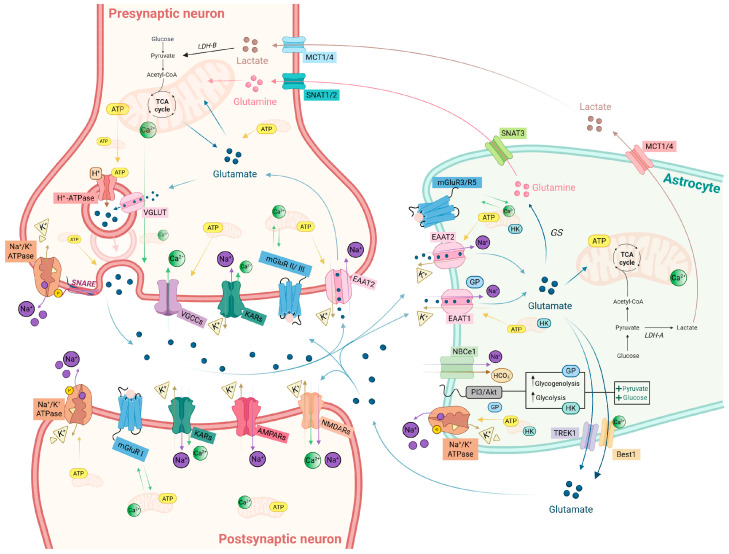
Tripartite synapse and glutamatergic transmission with compartment-specific metabolic support. Schematic representation of a tripartite glutamatergic synapse, depicting the presynaptic terminal and postsynaptic neuron (left) and the surrounding astrocyte (right). In the presynaptic terminal, action potential–evoked Ca^2+^ entry through voltage-gated Ca^2+^ channels (VGCCs) triggers soluble N-ethylmaleimide-sensitive factor attachment protein receptor (SNARE)-dependent exocytosis of glutamate-filled synaptic vesicles. Vesicles are loaded by vesicular glutamate transporters (VGLUTs) using a proton gradient generated by the vesicular H^+^-ATPase. Presynaptic mitochondria provide ATP for Na^+^/K^+^-ATPase activity, vesicle cycling, and Ca^2+^ clearance and sustain local tricarboxylic acid (TCA) cycle metabolism. Released glutamate activates ionotropic AMPA, NMDA and kainate receptors (AMPARs, NMDARs, and KARs), as well as group I metabotropic glutamate receptors (mGluR1/5) on the postsynaptic neuron, driving Na^+^ and Ca^2+^ influx, K^+^ efflux, and downstream dendritic signaling supported by postsynaptic mitochondria. Perisynaptic astrocytic processes express high-affinity excitatory amino acid transporters (EAATs), including EAAT1 (also known as glutamate–aspartate transporter, GLAST) and EAAT2 (also known as glutamate transporter-1, GLT-1), which clear glutamate from the synaptic cleft through Na^+^/K^+^-dependent, ATP-consuming uptake coupled to Na^+^/K^+^-ATPase and the Na^+^/HCO_3_^−^ electrogenic Na^+^/HCO_3_^−^ cotransporter 1 (NBCe1). Internalized glutamate is converted to glutamine by glutamine synthetase (GS) and returned to neurons via sodium-coupled neutral amino acid transporters (SNATs), including SNAT1/2 in neurons and SNAT3 in astrocytes, closing the glutamate–glutamine cycle. Astrocytes meet the energetic demands of glutamate uptake through glycolysis and glycogenolysis, regulated by hexokinase (HK), glycogen phosphorylase (GP), and phosphoinositide 3-kinase (PI3K)/protein kinase B (Akt) signaling. Pyruvate is converted to lactate by lactate dehydrogenase A (LDH-A); lactate is shuttled to neurons through monocarboxylate transporters (MCT1/4), where lactate dehydrogenase B (LDH-B) reconverts it to pyruvate to fuel the neuronal TCA cycle (astrocyte–neuron lactate shuttle, ANLS). Astrocytes also express mGluR3 and mGluR5, two-pore domain potassium channels (e.g., TWIK-related K^+^ channel-1, TREK1) and the Ca^2+^-activated anion channel bestrophin-1 (Best1), which contribute to K^+^ buffering, Ca^2+^ signaling, and gliotransmitter release. Colors and symbols highlight key species and pathways: glutamate (dark blue), glutamine (pink), lactate (brown), ATP (yellow), Ca^2+^ (green), Na^+^ (purple), K^+^ (light yellow), and mitochondria (stylized organelles in each compartment). Directional arrows indicate molecular or signaling fluxes, while arrows marked with ↑ indicate increased activity of the corresponding processes.

A detailed overview of glutamate handling and its compartment-specific metabolic support at the tripartite synapse is provided in Figure 1.

In this Review, we synthesize how mitochondrial bioenergetics, Ca^2+^ dynamics, redox signaling, and organelle positioning coordinate glutamate handling at the tripartite synapse; how their disruption is proposed to progressively shift excitatory signaling from physiological excitability toward hyperexcitability and excitotoxicity; and why AD represents a compelling model of a mitochondria-driven glutamatergic synaptopathy.

## 2. Mitochondrial Ca^2+^ Signaling at the Crossroads of Excitation and Metabolism

**Figure 2:**
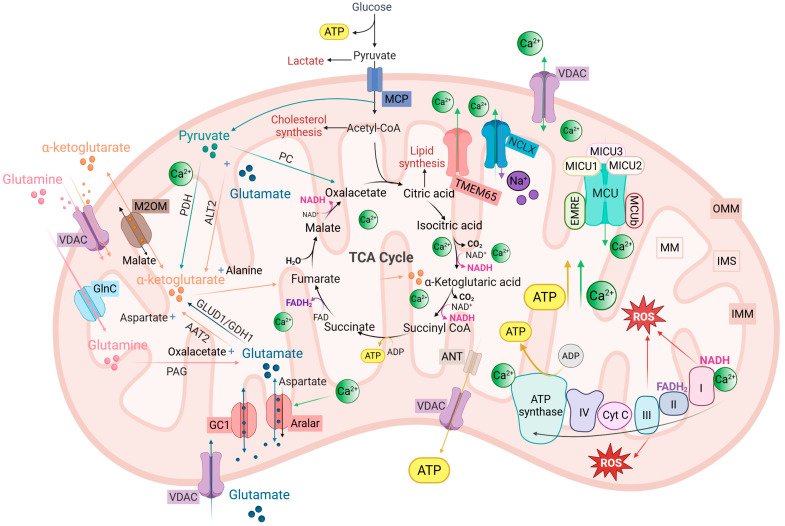
Mitochondrial architecture and Ca^2+^-metabolic integration at the core of glutamate handling. Schematic representation of a mitochondrion highlighting the outer mitochondrial membrane (OMM), inner mitochondrial membrane (IMM), intermembrane space (IMS), and matrix. The diagram integrates Ca^2+^ transport pathways with glutamate/glutamine metabolism, the tricarboxylic acid (TCA) cycle, and oxidative phosphorylation (OXPHOS). Glucose-derived pyruvate enters the matrix via the mitochondrial pyruvate carrier (MPC) and fuels the TCA cycle through pyruvate dehydrogenase (PDH) or pyruvate carboxylase (PC), while alanine aminotransferase 2 (ALT2) links pyruvate to amino acid metabolism. Glutamine is imported via a putative mitochondrial glutamine transport pathway (GlnC) and converted to glutamate by phosphate-activated glutaminase (PAG). Glutamate is transported across the IMM by glutamate carrier 1 (GC1; SLC25A22) and the Ca^2+^-regulated aspartate–glutamate carrier (Aralar/AGC1) and is further metabolized by glutamate dehydrogenase (GLUD1/GDH1) and/or mitochondrial aspartate aminotransferase (GOT2/AAT2) to generate α-ketoglutarate (α-KG), aspartate, and oxaloacetate, thereby feeding the TCA cycle and the malate–aspartate shuttle together with the malate–2-oxoglutarate carrier (M2OM). TCA cycle activity produces NADH and FADH_2_, which drive electron flow through complexes I–IV of the electron transport chain (ETC) and ATP synthesis via ATP synthase. Adenine nucleotides are exchanged by the adenine nucleotide translocator (ANT), and ATP/ADP fluxes to the cytosol occur through voltage-dependent anion channels (VDAC). Ca^2+^ entry through VDAC at the OMM and the mitochondrial Ca^2+^ uniporter (MCU) complex at the IMM—comprising MCU, its regulatory subunits MICU1/2/3, EMRE, and the modulatory subunit MCUb—is balanced by Ca^2+^ extrusion via the mitochondrial Na^+^/Ca^2+^ exchanger (NCLX). TMEM65 denotes an IMM transmembrane protein proposed to contribute to mitochondrial Ca^2+^ extrusion. Matrix Ca^2+^ stimulates TCA cycle dehydrogenases and ATP synthase, whereas excessive Ca^2+^ uptake and elevated electron transport can enhance reactive oxygen species (ROS) production. Colors and symbols highlight major species and pathways: Ca^2+^ (green), ATP (yellow), glutamate (dark blue), glutamine (pink), α-KG (orange), TCA intermediates (black), and transporters/enzymes (colored membrane proteins). Colored arrows trace the metabolic fate of specific substrates or molecules, whereas black arrows indicate the canonical reactions of the TCA cycle.

In neurons and astrocytes, mitochondria are central regulators of intracellular Ca^2+^ homeostasis. They sense local Ca^2+^ fluctuations and convert them into metabolic and redox responses that tune cellular activity. The main components of this mitochondrial Ca^2+^-metabolic axis—including mitochondrial Ca^2+^ machinery, glutamate/glutamine handling, the TCA cycle, and the respiratory chain—are schematized in Figure 2 and summarized in Table 1.

The ability of mitochondria to accumulate Ca^2+^ in a respiration- and membrane potential-dependent manner was first demonstrated in the early 1960s [43,44]. This phenomenon was mechanistically clarified with the discovery of the mitochondrial Ca^2+^ uniporter (MCU) and its regulatory subunits MICU1/2/3 and MCUb, which together form a Ca^2+^-gated channel complex in the inner mitochondrial membrane (IMM) [45,46] (reviewed in [47]).

Cytosolic Ca^2+^ signals arise through multiple routes—including voltage-gated, store-operated, and receptor-operated Ca^2+^ channels—as well as endoplasmic reticulum (ER) release via inositol 1,4,5-trisphosphate (IP_3_) receptors (IP_3_Rs) and ryanodine receptors (RyRs). These signals are terminated by the coordinated action of plasma membrane Ca^2+^-ATPase (PMCA), the sarco/endoplasmic reticulum Ca^2+^-ATPase (SERCA), and Na^+^/Ca^2+^ exchangers (NCX) [48,49]. The amplitude, frequency, and spatial profile of cytosolic Ca^2+^ changes are therefore shaped not only by the kinetics of Ca^2+^ entry and removal but also by the positioning and uptake capacity of mitochondria, which act as dynamic buffers embedded within these signaling networks. By buffering Ca^2+^ microdomains and coupling them to metabolism, mitochondria tune the impact of synaptic activity on cellular energy balance.

To reach the mitochondrial matrix, Ca^2+^ must cross both mitochondrial membranes. The outer mitochondrial membrane (OMM), which is relatively permeable to ions and metabolites via voltage-dependent anion channels (VDACs), allows rapid equilibration between cytosol and intermembrane space. The IMM, by contrast, forms the main regulatory barrier. Here, Ca^2+^ enters through the MCU complex, driven by the large negative membrane potential (ΔΨm ≈ −150 to −180 mV), and is tightly gated by MICU proteins, which prevent uptake at resting Ca^2+^ levels and permit it only when local Ca^2+^ rises transiently [50]. Early biochemical studies suggested that MCU opening requires cytosolic Ca^2+^ concentrations in the 10–20 µM range [51], far higher than those measured during global Ca^2+^ signals in intact cells. This apparent discrepancy led to the Ca^2+^ microdomain hypothesis, which proposes that mitochondria are strategically positioned at nanoscale contact sites with the ER or plasma membrane (PM), where local Ca^2+^ can transiently reach tens of micromolar concentrations sufficient to activate the low-affinity MCU [52,53]. For example, at mitochondria–ER contact sites (MERCs), ER Ca^2+^ release channels are aligned with VDAC and the MCU complex across the two membranes, minimizing diffusion distance and coupling ER Ca^2+^ fluxes to mitochondrial Ca^2+^ entry [54,55]. While such privileged contacts are well supported, the exact peak Ca^2+^ amplitudes, their duration, and the extent to which this quantitative regime generalizes across cell types and subcellular compartments remain active areas of investigation.

Mitochondria are not designed to store Ca^2+^; sustained matrix accumulation is intrinsically hazardous. Ca^2+^ entry via MCU must be continuously counterbalanced by efflux, primarily via the Na^+^/Ca^2+^ exchanger (NCLX), which extrudes Ca^2+^ from the matrix to the intermembrane space in exchange for Na^+^, allowing Ca^2+^ to diffuse back into the cytosol. Additional efflux mechanisms include Ca^2+^/H^+^ exchange mediated by TMBIM5 (transmembrane Bax inhibitor motif-containing protein 5) [56], and recent evidence suggests that TMEM65 (transmembrane protein 65) may also contribute to mitochondrial Na^+^/Ca^2+^ exchange [57,58,59,60]. Importantly, NCLX is currently the best-established efflux route in neurons, whereas the relative contribution, molecular mechanism, and context dependence of TMBIM5- and TMEM65-linked pathways remain less resolved. Together, rapid uptake coupled to a comparatively slower release enables mitochondria to sculpt the amplitude and duration of cytosolic Ca^2+^ signals [49].

When Ca^2+^ influx exceeds efflux capacity, however, mitochondrial function shifts from adaptive to pathological. Matrix overload favors opening of the mitochondrial permeability transition pore (mPTP), a high-conductance channel whose activation collapses ΔΨm, dissipates ion gradients, and can induce mitochondrial swelling and OMM rupture, with release of pro-death factors. Cyclophilin D and complexes involving ATP synthase and/or the adenine nucleotide translocase (ANT) have been implicated in regulating and/or forming the mPTP, although its precise molecular identity remains debated [61].

Taken together, mitochondrial Ca^2+^ handling operates along a continuum: transient microdomain-restricted Ca^2+^ uptake supports metabolism and redox signaling, whereas prolonged or excessive influx promote ΔΨm collapse, mPTP opening and downstream injury. Thus, mitochondrial Ca^2+^ signaling operates within a narrow physiological window, supporting metabolism at low-to-moderate loads but precipitating dysfunction when Ca^2+^ influx is excessive or prolonged.

### 2.1. Mitochondrial Ca^2+^ Signaling at the Glutamatergic Synapse: The Neuronal Perspective

In glutamatergic neurons, the general principles of mitochondrial Ca^2+^ handling are tuned to the unique demands of fast synaptic transmission. Even modest increases in global cytosolic Ca^2+^ (e.g., from ~100 nM to ~200–300 nM) produce local Ca^2+^ microdomains that are thought to drive rapid mitochondrial Ca^2+^ uptake, leading to large increases in total matrix Ca^2+^, while free matrix Ca^2+^ remains buffered in the low micromolar range due to Ca^2+^–phosphate buffering and slower NCLX-mediated efflux [62,63,64,65,66,67,68,69,70]. Importantly, neuronal mitochondria buffer not only activity-driven Ca^2+^ signals but also constitutive Ca^2+^ influx and ER Ca^2+^ leak, with RyR-mediated ER Ca^2+^ release representing a major source of mitochondrial Ca^2+^ in several neuronal populations [71,72].

Neuronal mitochondria are not functionally uniform. Synaptic mitochondria located within presynaptic boutons and near postsynaptic specializations show greater sensitivity to Ca^2+^ signals and greater vulnerability to Ca^2+^ overload than their non-synaptic counterparts [73]. This heterogeneity likely reflects molecular specialization of the MCU complex: MCU forms the pore, while MICU1/2, the neuron-enriched MICU3, and the inhibitory paralogue MCUb tune the activation threshold and dynamic range of mitochondrial Ca^2+^ uptake [45,46,47]. MICU3 is particularly important: enriched in axons and presynaptic boutons, it lowers the Ca^2+^ threshold for MCU opening, enabling mitochondria to respond to modest physiological Ca^2+^ elevations and to support activity-dependent ATP production at excitatory terminals [74]. Proteomic profiling further reveals pronounced cell type- and compartment-specific remodeling of mitochondrial proteins—including MCU-complex components, MERC tethers, and metabolic enzymes consistent with the idea that mitochondrial Ca^2+^ homeostasis is matched to neuronal identity and synaptic location [75].

Loss- and gain-of-function studies illustrate how mitochondrial Ca^2+^ handling operates within a narrow “safe window”. Mitochondria isolated from MCU knockout mice have been reported to retain a residual, Ru360-sensitive (i.e., inhibited by Ru360, a selective pharmacological blocker of the MCU) mitochondrial Ca^2+^ uptake capacity [76] and show intact baseline motor and cognitive performance [77], suggesting the existence of compensatory, low-capacity Ca^2+^-uptake pathways [76]. Under physiological conditions, these pathways appear sufficient; under stress, however, MCU function becomes increasingly important. Inhibition of MCU protects neurons by limiting Ca^2+^-driven mitochondrial depolarization and preventing cell loss in models of hypoxic–ischemic damage and diabetic neuropathy [78,79]. Conversely, intact MCU-dependent uptake is required for axonal maintenance and remyelination during inflammatory challenge [80].

Manipulations of MCU expression further support the concept of a finely tuned operating window: partial MCU knockdown mitigates glutamate-induced mitochondrial failure and excitotoxicity, whereas MCU overexpression alone is sufficient to trigger mitochondrial dysfunction and neuronal degeneration [81,82,83]. This balance is enforced by regulatory subunits: MICU1, the principal gatekeeper, prevents MCU opening at low cytosolic Ca^2+^; its loss causes chronic mitochondrial Ca^2+^ overload, synaptic dysfunction, and neurodegeneration [84,85,86,87], while MCUb dampens Ca^2+^ entry, and its deletion lowers the threshold for depolarization under glutamate stress in a sex-dependent manner [88,89].

Within the physiological window defined by these regulators, mitochondrial Ca^2+^ signals couple excitation to metabolism through two coordinated modules. High-amplitude or sustained Ca^2+^ entry engages matrix dehydrogenases to boost TCA cycle flux and OXPHOS [90,91], whereas lower-amplitude oscillations activate Ca^2+^-responsive carriers and the Aralar/aspartate-glutamate carrier 1 (AGC1)-dependent malate–aspartate shuttle, sustaining basal respiration, preserving NAD^+^/NADH balance, and supporting glycolysis [91,92]. These Ca^2+^-dependent mechanisms together align metabolic output with firing rate and are thought to contribute to the stability of neuronal excitability [91].

Balanced Ca^2+^ signaling also requires efficient Ca^2+^ efflux. NCLX, the most studied mitochondrial Na^+^/Ca^2+^ exchanger, mediates matrix Ca^2+^ extrusion and is essential for neuronal survival. In *C. elegans*, the orthologue NCX-9 regulates mitochondrial Ca^2+^ efflux and axon guidance [91,93], while in humans, loss-of-function mutations in SLC8B1/NCLX cause severe intellectual disability. In mice, NCLX deletion causes mitochondrial Ca^2+^ overload and depolarization, impairs presynaptic Ca^2+^ transients, reduces glutamate release probability, and abolishes hippocampal LTP [94]. NCLX activity is controlled by phosphodiesterase 2 (PDE2)–cyclic AMP (cAMP)–protein kinase A (PKA) signaling and is required for neuroprotection and hippocampus-dependent learning [95]. Conversely, NCLX downregulation alone can convert physiological synaptic activity into a lethal Ca^2+^ stimulus and trigger neuroglial degeneration [96].

TMEM65 remains relatively understudied, but recent work implicates it in mitochondrial Ca^2+^ efflux, either as a component of mitochondrial Na^+^/Ca^2+^ exchange or as a regulator of NCLX-dependent Ca^2+^ extrusion. Consistent with an essential role in mitochondrial Ca^2+^ homeostasis, biallelic pathogenic TMEM65 variants have been linked to severe mitochondrial disease with neurological involvement, and loss of *Tmem65* in vivo produces profound neurodevelopmental and excitability phenotypes [57].

Taken together, these findings support a framework in which MCU-mediated Ca^2+^ uptake and NCLX/TMEM65-dependent efflux define the physiological operating range of mitochondrial Ca^2+^ cycling in neurons. Within this range, synaptic mitochondria translate patterned Ca^2+^ signals into adaptive metabolic and synaptic responses that support glutamatergic function. Outside this range, experimental evidence indicates that the same machinery precipitates mitochondrial collapse, excitotoxicity, and degeneration. Thus, neuronal mitochondrial Ca^2+^ cycling stabilizes synaptic transmission and plasticity when balanced, but accelerates excitotoxic vulnerability when this balance is lost.

#### 2.1.1. Mitochondrial Ca^2+^ Signaling at the Presynapse

At glutamatergic terminals, mitochondria cluster near VGCCs and active zones, placing them near the Ca^2+^ microdomains generated during synaptic activity [97,98].

From this privileged position, presynaptic mitochondria sense activity-evoked Ca^2+^ elevations to tune release probability, balance vesicle cycling, and match ATP production to synaptic demand. When mitochondrial Ca^2+^ uptake is compromised, presynaptic Ca^2+^ transients become larger, asynchronous, and delayed release increases, synaptic depression accelerates, and high-frequency transmission becomes unstable [99,100,101,102]. Conversely, a moderate reduction in MCU activity can enhance vesicle fusion at mitochondria-containing boutons and facilitate mossy fiber LTP, suggesting that the optimal degree of mitochondrial Ca^2+^ uptake is synapse-specific and finely tuned rather than simply maximal [103].

Under repetitive high-frequency firing, rapid mitochondrial Ca^2+^ uptake combined with slower NCLX-dependent efflux generates a residual elevation of matrix Ca^2+^. This prolonged mitochondrial Ca^2+^ signal has been proposed to contribute to post-tetanic potentiation, thereby linking presynaptic mitochondrial Ca^2+^ handling to short-term plasticity [99,104,105,106]. Disruption of mitochondrial positioning—through fragmentation, impaired transport, or loss of docking—breaks this coupling, leading to increased asynchronous release, premature vesicle pool depletion, and reduced synaptic fidelity during sustained activity [107,108,109,110].

Beyond Ca^2+^ buffering, presynaptic mitochondria play a key bioenergetic role. Action potential–evoked Ca^2+^ entry into presynaptic mitochondria activates Ca^2+^-sensitive dehydrogenases and can enhance ATP synthesis efficiency, thereby boosting OXPHOS proportionally to synaptic workload [18,111,112,113]. At low firing rates, glycolysis and ATP diffusion from neighboring mitochondria are sufficient to sustain neurotransmitter release [114]. However, during intense stimulation, mitochondrial ATP production becomes rate-limiting. *Drosophila* mutants for *Miro* (Mitochondrial Rho GTPase), *Drp1* (Dynamin-related protein 1), and *Marf* (Mitochondrial assembly regulatory factor, the Mitofusin orthologue) illustrate this principle: impaired mitochondrial recruitment or dynamics disrupt reserve-pool mobilization and reduce the capacity for sustained neurotransmission [115,116,117]. In mammalian boutons, activity-dependent recruitment of glucose transporter 4 and glucose-dependent glycolysis supports neurotransmission, whereas conditions that force reliance on lactate/pyruvate reveal a strict requirement for MCU-dependent OXPHOS to sustain endocytosis and vesicle recovery [74,107,118,119,120]. Consistently, neuronal MCU deletion disrupts metabolic support for high-frequency firing and impairs gamma oscillations and sharp-wave ripples ex vivo and in vivo, underscoring the central role of presynaptic mitochondrial Ca^2+^ uptake in network-level synchrony [121].

Overall, presynaptic mitochondrial Ca^2+^ handling plays a central role in regulating release timing, vesicle availability, and the metabolic support required for high-frequency transmission. When Ca^2+^ influx persistently exceeds mitochondrial buffering and metabolic capacity, experimental evidence indicates that presynaptic mitochondria can depolarize and engage pro-death signaling (including cytochrome c release), thereby increasing the susceptibility of highly active glutamatergic terminals to excitotoxic injury. In summary, presynaptic mitochondria improve the timing and sustainability of glutamate release by buffering Ca^2+^ microdomains and supplying ATP; Ca^2+^ overload flips this benefit into a liability.

#### 2.1.2. Mitochondrial Ca^2+^ Signaling in Soma and Axon

In glutamatergic neurons, somatic and axonal mitochondria integrate Ca^2+^ signals arising across much larger spatial domains than those encountered at individual synapses. These organelles sense Ca^2+^ influx through VGCCs as well as Ca^2+^ released from intracellular stores, buffer local and global cytosolic Ca^2+^ elevations, and prevent sustained increases that would compromise excitability. By restricting perisomatic and axonal Ca^2+^ levels, mitochondria limit the activation of Ca^2+^-activated K^+^ channels—particularly large- and small-conductance Ca^2+^-activated K^+^ channels (BK and SK, respectively)—and reduce Ca^2+^-dependent inactivation of VGCCs, thereby stabilizing firing patterns and maintaining spike-frequency adaptation [122,123].

Along myelinated axons and at nodes of Ranvier, mitochondria are strategically positioned to sustain reliable action potential conduction. Their ATP production supports Na^+^/K^+^-ATPase activity during repetitive firing, while their buffering of activity-evoked Ca^2+^ influx prevents local Ca^2+^ overload [80]. Mitochondrial transport, docking and immobilization are themselves Ca^2+^-regulated processes, enabling mitochondria to accumulate at high-demand sites as neuronal activity increases [107,108,109,110].

In neuronal somata, high mitochondrial density facilitates fine control of Ca^2+^ dynamics. One of the few in vivo studies to directly monitor mitochondrial Ca^2+^ in cortical neurons of awake mice revealed a “loose coupling” between cytosolic and mitochondrial Ca^2+^ transients: mitochondria were recruited in a probabilistic manner and showed increased coupling fidelity during behavioral activation in a Ca^2+^/calmodulin-dependent protein kinase II (CaMKII)-dependent manner [124].

Together, these mechanisms establish somatic and axonal mitochondria as dynamic integrators of neuronal workload and stress. By selectively coupling spatially constrained Ca^2+^ signals to mitochondrial metabolism, they stabilize firing patterns, support sustained activity, and are thought to contribute to the maintenance of network oscillations [121].

#### 2.1.3. Mitochondrial Ca^2+^ Signaling at the Postsynapse

In glutamatergic neurons, postsynaptic mitochondria are enriched in dendritic shafts and at the base of spines, positioning them in proximity to Ca^2+^ influx domains generated by NMDARs and VGCCs, where they sample highly localized Ca^2+^ microdomains. By rapidly sequestering activity-evoked Ca^2+^ and releasing it back more slowly via mitochondrial Ca^2+^ efflux pathways, they restrict lateral Ca^2+^ diffusion, limit crosstalk between neighboring spines, and maintain biochemical compartmentalization over distances of only a few micrometers [63,125,126,127]. At rest, mitochondrial Ca^2+^ levels remain close to cytosolic concentrations (~100 nM), whereas synaptic depolarization elicits mitochondrial Ca^2+^ transients that occur with higher probability at spine bases than along dendritic shafts. This spatial bias suggests that postsynaptic mitochondria decode not only the intensity but also the spatial origin of incoming excitatory input, preferentially coupling mitochondrial activation with local synaptic input [63,125].

This coupling is further refined at the level of neuronal identity and dendritic domains. In hippocampal Cornu Ammonis 2 (CA2) neurons, MCU expression is selectively enriched in distal dendrites, regions that receive distinct inputs and can express specialized forms of plasticity. Local MCU loss diminishes mitochondrial Ca^2+^ uptake, impairs distal synaptic plasticity, and is associated with mitochondrial fragmentation and reduced spine head size, suggesting that compartment-specific MCU expression aligns postsynaptic mitochondrial performance with synaptic demands [128].

By linking Ca^2+^ entry to oxidative metabolism, postsynaptic mitochondria couple excitatory synaptic activity to the energetic and structural demands of LTP. Ca^2+^-dependent activation of TCA cycle dehydrogenases transiently boosts ATP production in response to NMDAR activation and burst firing, providing energy for AMPAR and NMDAR trafficking, actin cytoskeleton remodeling, and de novo protein synthesis—processes essential for the stabilization of LTP and spine structural changes [129,130,131].

Together, these findings indicate that postsynaptic mitochondrial Ca^2+^ signaling links local excitatory input to the energetic and structural processes required for synaptic plasticity. Disruption of this coupling has been proposed to compromise synaptic stability and increase vulnerability to degenerative stress.

### 2.2. Mitochondrial Ca^2+^ Signaling at the Glutamatergic Synapse: The Astrocytic Perspective

Although not electrically excitable in the classical sense, astrocytes are now recognized as integral computational elements of neuronal circuits. Their elaborate morphology enables them to act as physical and functional bridges, translating synaptic activity into Ca^2+^ signals with defined spatial scales, amplitudes, and frequencies [4,132,133,134,135,136,137].

Astrocytic Ca^2+^ dynamics differ fundamentally from those in neurons. Neurons communicate through fast, all-or-none electrical spikes that evoke millisecond Ca^2+^ transients, whereas astrocytes generate a slower, more diverse repertoire of Ca^2+^ events [133,134,135,137]. These include highly localized microdomains in PAPs, branch- and soma-level oscillations, and long-range intercellular waves [137,138,139,140,141,142]. Together, this multi-layered signaling is thought to enable astrocytes to integrate synaptic input with neuromodulatory tone and metabolic cues [134,137,143,144,145,146,147,148].

Astrocytes rely on multiple mechanisms to generate cytosolic Ca^2+^ elevations. Metabotropic pathways activated by G protein-coupled receptors (GPCRs), primarily Gq-coupled receptors—including mGluRs and P2Y receptors—stimulate phospholipase C, leading to IP_3_ production and IP_3_R-mediated ER Ca^2+^ release, while Gi-coupled receptors can modulate Ca^2+^ signaling indirectly in specific contexts [133,134,137,146,149]. Astrocytes also mobilize Ca^2+^ through PM pathways, including reverse-mode Na^+^/Ca^2+^ exchange, transient receptor potential (TRP) channels, ionotropic receptors, and, under certain conditions, VGCCs [138,141,146]. In addition, store-operated Ca^2+^ entry (SOCE), triggered by ER Ca^2+^ depletion and mediated by stromal interaction molecule (STIM)-Orai signaling at ER-PM junctions, provides sustained Ca^2+^ influx and supports ER refilling [137,146]. High-resolution imaging has revealed that most astrocytic Ca^2+^ activity arises in microdomains within distal processes, often independent of somatic Ca^2+^ changes and driven either spontaneously or by local synaptic input [137,138,139,140,141,142,150]. These microdomains exhibit considerable heterogeneity in amplitude, duration, and spatial spread, reflecting the diverse morphology and molecular composition of PAPs [135,137,140,150,151]. Their properties are further shaped by neuromodulators, metabolic state, and the extracellular milieu [146,150,151,152,153].

Astrocytes also generate robust spontaneous Ca^2+^ activity even when neuronal firing is silenced, pointing to an intrinsic astrocytic “excitability” driven by stochastic Ca^2+^ fluxes through PM channels and intracellular stores [138,140,142,154]. These spontaneous microdomains are not mere noise: recent work shows that spontaneous, stochastic Ca^2+^ microdomains in PAPs are functional signals required for LTP and memory retention [155].

Mitochondria emerge as key regulators of this intrinsic Ca^2+^ activity. Transient mPTP openings have been proposed to contribute to highly localized cytosolic Ca^2+^ microdomains with event frequency enhanced by neuronal activity and ROS production [150]. While several studies support this model, the extent to which mPTP “flickers” represents a general signaling mechanism remains unresolved. In addition, mitochondria can tune the amplitude and spatial spread of spontaneous events by dynamically adjusting their Ca^2+^ uptake capacity [156].

Microdomain Ca^2+^ signals enable astrocytes to integrate information from thousands of synapses into highly compartmentalized responses that regulate gliotransmission, K^+^ and glutamate homeostasis, neurovascular coupling, and structural remodeling of PAPs. By rapidly sequestering Ca^2+^, mitochondria contribute to defining the spatial boundaries of microdomains and stabilizing local cytosolic Ca^2+^ dynamics [157].

Recent work [158] further suggests that many PAPs may be organized into “leaflet” domains that envelop clusters of synapses and may function as multisynaptic integrators. Conceptually, leaflet domains could enable spatially restricted Ca^2+^ computations over groups of neighboring synapses, rather than single-synapse units. Ultrastructural analyses show that leaflet tips frequently contain ER but lack mitochondria while remaining continuous with mitochondria-rich astrocytic shafts. This architecture suggests that mitochondrial Ca^2+^ handling in adjoining compartments may indirectly constrain leaflet Ca^2+^ microdomains and support the energetic demands associated with local glutamate uptake. Overall, astrocytic mitochondria act as spatial regulators of perisynaptic Ca^2+^ microdomains and couple local Ca^2+^ signals to glutamate uptake and metabolic support.

#### 2.2.1. Mitochondrial Ca^2+^ and Control of Astrocytic Microdomains

Astrocytic mitochondria act as dynamic Ca^2+^ buffers: they take up Ca^2+^ through the MCU, driven by the large negative ΔΨm, and release it back to the cytosol primarily via NCLX [156,157]. Early imaging studies showed that ER-driven cytosolic Ca^2+^ rises are followed by mitochondrial Ca^2+^ uptake, and that dissipation of ΔΨm with FCCP (carbonyl cyanide-p-trifluoromethoxyphenylhydrazone) prolongs cytosolic Ca^2+^ decay, highlighting a major contribution of mitochondria in Ca^2+^ clearance [157]. Disrupting this buffering capacity can dramatically alter astrocytic Ca^2+^ signaling: MCU inhibition amplifies cytosolic Ca^2+^ transients and can enhance Ca^2+^-dependent gliotransmitter release, whereas blocking NCLX or mPTP suppresses Ca^2+^-dependent astrocyte-to-neuron signaling [159]. Notably, astrocytic mitochondria appear to exhibit a higher Ca^2+^ buffering capacity than their neuronal counterparts [160], consistent with their role in sustaining prolonged, integrative Ca^2+^ signals rather than brief spikes.

In addition to ER release, PM pathways provide a major source of Ca^2+^ for astrocytic mitochondria. SOCE is activated when ER Ca^2+^ stores are depleted: STIM proteins sense the drop in luminal Ca^2+^ and activate Orai channels (and, in some contexts, TRP channels), leading to sustained Ca^2+^ influx that is efficiently taken up by nearby mitochondria [138,143,149,161]. Silencing NCLX reduces both the amplitude and rate of SOCE, indicating tight coupling between mitochondrial buffering capacity and PM Ca^2+^ entry [162].

At a structural level, the efficiency of Ca^2+^ exchange between mitochondria and the ER or PM is determined by the nanoscale spacing between these membranes. Artificial manipulation of the MERC distance has shown that both excessive separation and excessive proximity disrupt Ca^2+^ transfer [163]. In astrocytes, such privileged interfaces are abundant in PAP-associated domains containing both mitochondria and ER [164], positioning mitochondria to read out and shape local Ca^2+^ microdomains that control glutamate transport and astrocyte-mediated synaptic modulation.

Astrocytic mitochondria exhibit slow, bidirectional transport along fine processes and are unevenly distributed within PAP-associated domains, differing markedly from neuronal mitochondrial behavior [165]. Both microtubules and actin filaments contribute to this trafficking, in contrast to neurons, where long-range movement is primarily microtubule-based [165,166]. While astrocytes express kinesin motors (notably kinesin-1 family members, e.g., KIF5 isoforms) and dynein, together with actin-based myosin, the mechanisms governing mitochondrial positioning within PAP-associated domains remain largely unexplored compared to the well-defined Miro/TRAK (trafficking kinesin-binding) system in neurons.

At glutamatergic synapses, astrocytic mitochondria often reside near EAAT1/GLAST and EAAT2/GLT-1, as well as nearby MERCs, thereby coupling synaptic activity, mitochondrial Ca^2+^ uptake, and ATP production to glutamate clearance [165,167]. Glutamate uptake itself can regulate mitochondrial positioning: Na^+^ co-transport elevates intracellular Na^+^, which may favor reverse-mode NCX operation, producing rapid, spatially confined Ca^2+^ microdomains that have been proposed to arrest mitochondria near EAAT2/GLT-1 clusters [143,165]. Through this reciprocal coupling, mitochondrial positioning, Ca^2+^ buffering, and metabolic support are spatially aligned with glutamate uptake sites.

#### 2.2.2. Mitochondrial Ca^2+^-Dependent Metabolic Integration, Glutamate Handling, and Vulnerability

Local Ca^2+^ transients within PAPs provide a mechanism to match mitochondrial ATP production to highly localized energetic demands. Mitochondrial Ca^2+^ elevations activate Ca^2+^-sensitive dehydrogenases of the TCA cycle and support astrocytic glycogenolysis via glycogen phosphorylase (GP), thereby fueling glycolysis and oxidative metabolism [164]. This coupling is exemplified by transient mitochondrial “mitoflashes”—brief, quantal events associated with ROS transients and changes in ΔΨm that mark focal metabolic load and coincide with highly active Ca^2+^ microdomains [38].

Loss or dysfunction of astrocytic mitochondria in PAP-associated domains impairs glutamate uptake and metabolic support. In experimental models of ischemia, loss of mitochondria from PAP-associated domains has been reported to precede overt neuronal death, while preservation of mitochondrial motility and Ca^2+^ handling sustains glutamate clearance and neuronal viability [168]. More broadly, pathological conditions characterized by excessive glutamate release and energetic stress impose sustained demands on astrocytic mitochondrial Ca^2+^ handling, promoting Ca^2+^ overload, mPTP opening, loss of ΔΨm, and ATP depletion. These processes are thought to compromise glutamate uptake, promote extracellular glutamate accumulation, and amplify excitotoxicity at the tripartite synapse [169].

Overall, mitochondrial Ca^2+^ cycling integrates perisynaptic Ca^2+^ signaling with metabolic support and glutamate clearance, stabilizing astrocyte–neuron coupling under physiological conditions while conferring vulnerability during sustained metabolic or excitotoxic stress.

### 2.3. The Landscape of Glutamatergic Transmission: Energy, Metabolism, and Ca^2+^ Control

The previous sections have outlined how mitochondrial Ca^2+^ uptake and release shape neuronal excitability and plasticity across pre-, post-, and perisynaptic compartments. Here, we focus on how the same Ca^2+^ signals control the metabolic wiring that supports glutamatergic transmission.

In neurons and astrocytes, matrix Ca^2+^ acts on a defined set of targets—including pyruvate, isocitrate, and α-ketoglutarate (α-KG) dehydrogenases, as well as Ca^2+^-regulated mitochondrial carriers—to adjust TCA cycle flux, OXPHOS, redox balance, and substrate allocation during synaptic activity [8,55,90,91,170,171,172,173].

In glutamatergic neurons, activity-dependent Ca^2+^ microdomains generated by VGCCs and NMDARs (and by Ca^2+^-permeable AMPARs where present) can drive mitochondrial Ca^2+^ uptake, acutely enhancing matrix dehydrogenases and accelerating the TCA cycle [18,111,112,113]. This coupling aligns firing frequency with ATP availability and sets the capacity for sustained synaptic transmission [8,18,173]. In parallel, Ca^2+^-responsive elements of the malate–aspartate shuttle (MAS), such as the Aralar/AGC1 carrier, tune how efficiently cytosolic NADH generated by glycolysis is oxidized in mitochondria. This provides a Ca^2+^-dependent link between neuronal glucose utilization, redox state, and mitochondrial ATP production [91,92,174,175]. Through coordinated regulation of TCA flux and MAS efficiency, mitochondrial Ca^2+^ handling couples firing frequency to ATP availability and sets the capacity for sustained vesicle cycling and ionic homeostasis.

Consistent with accumulating evidence for substantial astrocytic oxidative capacity, astrocytic mitochondria contribute significantly to total TCA flux and support Na^+^/K^+^-ATPase activity in vivo, while glycolysis and glycogenolysis provide metabolic flexibility under fluctuating demand [176,177]. Local Ca^2+^ elevations in PAPs can promote glycogenolysis, and accelerate glycolysis [177,178]. Mitochondrial Ca^2+^ uptake then influences the extent to which pyruvate is oxidized in the TCA cycle versus reduced to lactate, embedding glutamate clearance within a Ca^2+^-sensitive metabolic circuit that allocates ATP production and lactate export according to synaptic demand [38,176,177].

Within this framework, the astrocyte–neuron lactate shuttle (ANLS) remains a useful conceptual model [36,171,177], although accumulating evidence supports a bidirectional and context-dependent exchange of lactate between astrocytes and neurons [174,176,179,180]. Accordingly, lactate trafficking should be viewed as a flexible coupling mode. Ca^2+^-dependent control of glycolysis, pyruvate oxidation, and MAS activity in both neurons and astrocytes renders this metabolic cooperation highly dynamic [176,180,181].

Once inside astrocytes, glutamate reaches a metabolic branch point strongly influenced by mitochondrial Ca^2+^ handling. One branch converts glutamate to glutamine via GS, an ATP-consuming process tightly coupled to glycogen metabolism and essential for neurotransmitter recycling [10,37,179]. The remaining glutamate exchanges with mitochondrial α-KG via VDACs and IMM carriers such as the 2-oxoglutarate/malate carrier and the proton-coupled glutamate carrier GC1 (SLC25A22) [182,183,184]. In the matrix, glutamate is converted to α-KG mainly by mitochondrial aspartate aminotransferase (GOT2) and glutamate dehydrogenase (GDH). Transamination predominates at low glutamate concentrations, whereas GDH-driven oxidative deamination rises steeply during high activity [179,182]. Because GOT2 operates within the Ca^2+^-regulated MAS and GDH activity reflects the Ca^2+^-tuned redox and energetic state of the matrix, mitochondrial Ca^2+^ uptake helps govern the balance between glutamate oxidation, glutamine synthesis, and lactate-oriented carbon recycling [37,179,184].

The resulting α-KG can follow three main fates: (i) it can be fully oxidized in the TCA cycle, generating NADH and FADH_2_ to fuel OXPHOS and provide the ATP needed to support high-affinity glutamate uptake, K^+^ clearance, and glutamine synthesis [37,179]; (ii) a fraction of α-KG–derived carbons exit the TCA cycle as malate or oxaloacetate and is converted to pyruvate, which can be oxidized (full recycling) or reduced to lactate and exported (partial recycling). Partial recycling increases steeply when extracellular glutamate rises and acts as a metabolic “safety valve” that prevents intracellular glutamate accumulation while maintaining uptake capacity [37,179,184]; and (iii) citrate, malate, or α-KG can be exported to support biosynthesis and NADPH production. These cataplerotic fluxes are balanced by astrocyte-specific anaplerosis via pyruvate carboxylase, which replenishes TCA intermediates and underlies the net production of glutamate and glutamine from glucose [37,184]. In each case, mitochondrial Ca^2+^ regulates the partitioning among these fates by modulating dehydrogenase activity, MAS flux, and mitochondrial redox balance.

In neurons, transmitter glutamate synthesis is likewise tightly coupled to mitochondrial Ca^2+^. Astrocyte-derived glutamine is imported via SNAT1/2 (sodium-coupled neutral amino acid transporters) and converted to glutamate by phosphate-activated glutaminase (PAG), a mitochondrial enzyme associated with the IMM [10,11,183]. Updated pseudo-MAS models, supported by isotope tracing and carrier studies, indicate that glutamine entry into the matrix and glutamate/aspartate export are mediated by carrier systems that are functionally integrated with OXPHOS and MAS [174,175,183,184]. Because glutamine-to-glutamate conversion perturbs matrix pH and redox state, the ability to stabilize these changes depends on Ca^2+^-regulated dehydrogenases and on mitochondrial Ca^2+^ handling via MCU and NCLX [91,170,174]. Thus, the maintenance of transmitter glutamate pools during high-frequency activity is set not only by substrate availability but also by the Ca^2+^-dependent tuning of TCA flux, MAS activity, and carrier-mediated export.

Taken together, these metabolic pathways delineate a Ca^2+^-dependent landscape in which mitochondrial positioning, TCA cycle velocity, glutamate partitioning (between glutamine synthesis, full oxidation, and pyruvate recycling), and the balance of anaplerotic and cataplerotic fluxes are all adjustable parameters. Matrix Ca^2+^—sensed through dehydrogenases, shuttles, and carriers—provides a common control signal that links patterns of glutamatergic activity to the long-term sustainability of synaptic transmission [90,91,170,171].

### 2.4. Mitochondrial Ca^2+^–ROS Axis at the Glutamatergic Synapse

Mitochondrial Ca^2+^ signaling and ROS generation form an integrated signaling axis that links metabolic load to glutamatergic synapse function. Under physiological conditions, electron flow through the respiratory chain generates low-level superoxide and its more stable derivative H_2_O_2_, mainly at complexes I and III. These species are rapidly buffered by superoxide dismutases, peroxiredoxins, and glutathione-dependent systems, maintaining an “oxidative eustress” window in which H_2_O_2_ acts as a short-range signaling molecule rather than a source of damage [185,186].

During synaptic activity, activity-evoked Ca^2+^ entry is rapidly taken up by nearby mitochondria via the MCU complex. This stimulates Ca^2+^-sensitive dehydrogenases, boosting NADH supply and respiratory flux, and transiently increasing mitochondrial ROS production. Ca^2+^ extrusion via NCLX then contributes to restoring baseline matrix Ca^2+^ levels and redox tone [187,188]. At excitatory synapses, these brief Ca^2+^-evoked ROS pulses can reversibly oxidize cysteine residues on redox-sensitive targets—including glutamate receptors, transporters, ion channels, and kinases/phosphatases—thereby modulating channel gating, receptor trafficking, and the kinase–phosphatase balance that underlies synaptic plasticity [189,190].

In neurons, tightly packed respiratory supercomplexes minimize baseline electron leak, ensuring that physiological ROS levels remain low yet sufficient to fine-tune intrinsic excitability and synaptic plasticity. When Ca^2+^ overload or partial electron transport chain (ETC) inhibition over-reduces redox centers, ROS production is amplified, mitochondrial Ca^2+^ buffering fails, mPTP opening is favored, and excitotoxic cascades are triggered downstream of intense NMDAR or KAR activation [191,192].

In astrocytes, a less tightly assembled ETC architecture has been associated with a higher tonic mitochondrial ROS output that engages the nuclear factor erythroid 2–related factor 2 (Nrf2)–Kelch-like ECH-associated protein 1 (Keap1) axis and glutathione pathways, sustains EAAT2/GLT-1 expression and glutamate clearance. ROS has also been proposed to shape Ca^2+^ microdomains in PAPs via redox-sensitive mPTP “flickers” [151,193].

Together, mitochondrial Ca^2+^–ROS coupling provides a compartmentalized signaling framework that matches glutamatergic activity to metabolic capacity in neurons and astrocytes. While Ca^2+^-evoked mitochondrial redox signals are well supported, the precise spatiotemporal range, molecular targets, and compartment specificity of these ROS pulses at tripartite synapses are not fully resolved and likely depend on cell type, activity regime, and baseline metabolic state.

## 3. Excitotoxicity as an Engine of Neurodegeneration: Interconnected Roles of Glutamate, Mitochondria, and Hyperexcitability

In previous sections, we outlined how glutamate handling and mitochondrial Ca^2+^–metabolic coupling at the tripartite synapse support physiological signaling and synaptic plasticity. The same organizational principles, however, also frame how synapses fail. When astrocytic glutamate clearance, neuronal metabolic reserve, and mitochondrial Ca^2+^ handling become compromised, glutamatergic signaling progressively shifts from adaptive plasticity to neuronal hyperexcitability and, ultimately, glutamate-driven excitotoxicity. Several complementary frameworks have been proposed to explain glutamatergic dysregulation in disease, including receptor-centric models (e.g., preferential recruitment of eNMDAR signaling), excitation–inhibition (E/I) imbalance driven by interneuron vulnerability and inflammatory/microglia-mediated modulation of synaptic tone [42,194,195,196,197,198]. These frameworks are not mutually exclusive and likely operate in parallel, with their relative contribution varying across brain regions, disease stages, and activity regimes.

Neuronal hyperexcitability refers to a state in which neurons or networks are abnormally prone to generate action potentials—either in response to normally subthreshold inputs or as excessive spontaneous firing—due to imbalances between E/I and/or altered intrinsic membrane properties [42,199,200]. First recognized as a defining neurophysiological hallmark in epilepsy, cortical hyperexcitability is now recognized as an early and recurrent feature in a range of neurodevelopmental and neurodegenerative disorders, including AD (reviewed in [42]). At the cellular level, hyperexcitability translates into repeated bursts of glutamate release and sustained Ca^2+^ entry, imposing a chronic energetic and redox burden on mitochondria. As long as mitochondrial and glial safeguards can match this load, network hyperexcitability may remain functionally compensated; once these safeguards fail, the same glutamatergic drive is more likely to cross the threshold into excitotoxicity, with mitochondrial Ca^2+^ overload, bioenergetic collapse, and irreversible structural damage.

Excitotoxicity, classically defined as glutamate-driven neuronal death [201], can therefore be conceptualized not as a single catastrophic event but as the end point of a triad: glutamatergic dysregulation, mitochondrial failure, and neuronal hyperexcitability. While excessive glutamate receptor activation and Ca^2+^ overload in excitotoxic injury are well established, the relative contribution of upstream drivers (e.g., impaired astrocytic uptake, altered inhibition, inflammatory signaling) can vary across diseases, stages, and brain regions.

Within this triad, mitochondria emerge as key integrators. By coupling glutamate-dependent Ca^2+^ influx to ATP production, ROS generation, mPTP opening, and metabolic utilization of glutamate, they influence the excitotoxic threshold: initially sustaining high-frequency firing and ion homeostasis and, once bioenergetic reserve and Ca^2+^ buffering are exhausted, permitting irreversible Ca^2+^ deregulation and cell death [202]. Here, we build on these perspectives by proposing that mitochondrial Ca^2+^ handling and bioenergetic reserve act as convergent “threshold-setters” that determine whether increased glutamatergic drive remains reversible or progresses toward degeneration. In this context, excitotoxicity is not triggered by glutamate alone but emerges when glutamatergic load exceeds the buffering and metabolic capacity of astrocytes, neurons, and their mitochondria.

### 3.1. Cellular Determinants of Glutamate-Driven Hyperexcitability and Excitotoxicity

Glutamate-driven hyperexcitability and excitotoxicity arise as emergent properties of the neuron–astrocyte unit. Together, neurons and astrocytes set the threshold at which glutamatergic workload surpasses physiological limits through their coordinated control of glutamate clearance, metabolic support, and ion homeostasis.

**Figure 3:**
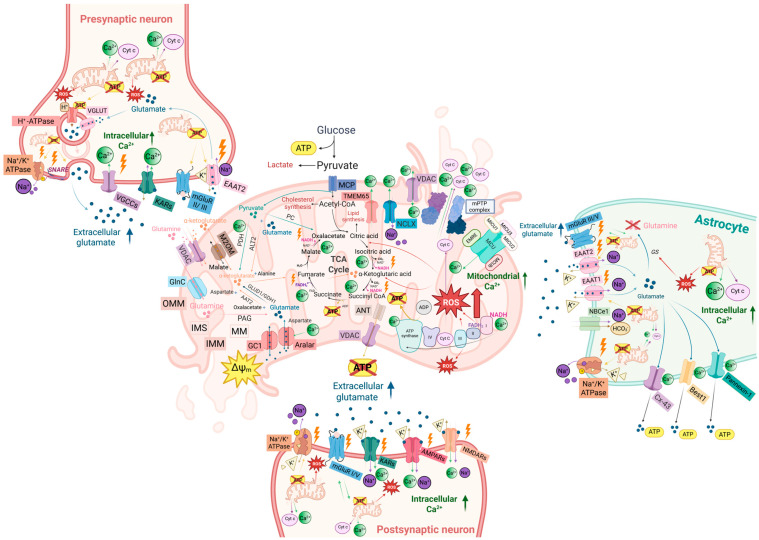
Mitochondrial dysfunction and glutamate-driven excitotoxicity. Schematic representation of a glutamatergic tripartite synapse under pathological conditions. Excessive glutamate release, together with impaired astrocytic uptake, elevates extracellular glutamate and drives persistent activation of pre- and postsynaptic glutamate receptors. The resulting intracellular Ca^2+^ overload imposes sustained metabolic and redox stress on neuronal and astrocytic mitochondria. In presynaptic, postsynaptic, and somatic neuronal mitochondria, excessive Ca^2+^ entry through the MCU complex and/or impaired Ca^2+^ extrusion (e.g., via NCLX) promote mPTP opening, loss of membrane potential (ΔΨm), increased ROS production, and cytochrome *c* release, leading to ATP depletion and further glutamate dysregulation. In astrocytes, oxidative damage to EAAT1/2, reduced GS activity, and altered Na^+^/K^+^-ATPase and NBCe1 function limit glutamate clearance and glutamine supply, while changes in mGluR3/5, Connexin-43, Best1, and TREK1 signaling contribute to abnormal Ca^2+^ dynamics and gliotransmission. The enlarged mitochondrion in the center provides a zoomed-in view of mitochondrial Ca^2+^ handling, metabolic pathways, and ROS production under excitotoxic stress. Colors highlight major species and events: glutamate (blue), glutamine (pink), Ca^2+^ (green), ATP (yellow), and ROS/ΔΨm loss/cytochrome *c* release (red). Colored arrows trace the direction and fate of specific substrates or molecules, whereas black arrows indicate canonical metabolic reactions; arrows denoting increases represent enhanced activity or accumulation of the indicated processes.

The main compartment-specific consequences of mitochondrial dysfunction and disrupted glutamate handling are illustrated in Figure 3 and summarized in Table 2.

#### 3.1.1. The Guardian Falls: Astroglial Dysfunction as a Driver of Hyperexcitability and Excitotoxicity

At tripartite synapses, astrocytes are the primary guardians of glutamate and K^+^ homeostasis. PAPs maintain low extracellular glutamate and K^+^ via high-affinity transporters and Na^+^/K^+^-dependent uptake, an energetically demanding task sustained by local mitochondria (see Section 2). The role of EAAT1/2-dependent glutamate uptake and its energetic coupling to Na^+^/K^+^-ATPase activity is well established as a first-line defense against spillover and excitotoxic receptor recruitment.

When this mitochondrial support falters, ATP-dependent glutamate uptake and K^+^ buffering decline, extracellular glutamate and K^+^ rise, and neurons become prone to hyperexcitability and excitotoxicity [203]. As PAPs retract and uptake slows, glutamate spills over to neighboring synapses and activates eNMDARs, elevating environmental glutamate and degrading the synaptic specificity required for synaptic plasticity [204,205,206].

Astrocytic metabolism further shapes excitability through the ANLS: activity-dependent glycolysis and oxidative metabolism generate lactate that fuels neuronal OXPHOS and modulates ion channel activity [36,207]. When astrocytic mitochondria are impaired, both glutamate-buffering capacity and metabolic support collapse, leaving neurons energy-deprived precisely when firing rates and glutamatergic load are highest. In disease states, reactive astrocytes do not simply lose homeostatic functions; they can actively promote hyperexcitability. Ca^2+^-dependent opening of Connexin-43 (Cx43) hemichannels, pannexin-1, and Bestrophin-1 (Best1) channels can drive non-vesicular release of glutamate, ATP, and D-serine, enhancing NMDAR activation and synchronizing network activity [208]. Because the relative contribution of these release pathways varies across preparations and pathological contexts, we frame reactive gliotransmission as a complementary amplifier mechanism whose impact is likely stage-, region-, and stimulus-dependent rather than uniform across conditions. Pro-inflammatory signaling—for example, tumor necrosis factor-α (TNF-α)–nuclear factor κB (NF-κB) activation—can further suppress EAAT2 expression and trafficking, weakening glutamate uptake and locking circuits into a vicious cycle of impaired clearance, reactive gliotransmission, and excitotoxic drive [209,210].

Thus, astrocytic mitochondria and PAPs act as a primary checkpoint for glutamate homeostasis: they power glutamate and K^+^ clearance, sustain lactate delivery to neurons, and tune Ca^2+^ and redox signals that govern gliotransmitter release, thereby determining whether astrocytes function as buffers or amplifiers of excitotoxic drive.

Overall, astrocytic mitochondrial support defines a first excitotoxic checkpoint by setting the efficiency of glutamate clearance and metabolic buffering at tripartite synapses.

#### 3.1.2. The Vulnerable Neuron: Bioenergetics and Hyperexcitability

Once the astroglial shield is weakened, neurons become the vulnerable second stage of the hyperexcitability–excitotoxicity cascade. Because ≈ 80% of neuronal ATP is devoted to Na^+^/K^+^-ATPase activity, neurons operate close to their metabolic limits [8]. Disruption of the ANLS removes a major source of rapidly available fuel and forces neurons to rely on their own, often compromised, glucose oxidation. The result is a bioenergetic “bottleneck” in which physiologically plausible synaptic activity can exceed available metabolic reserve. Intrinsic mitochondrial defects further intensify this vulnerability. In neurodegenerative models, impaired mitochondrial quality control and trafficking lead to the accumulation of depolarized, poorly functioning mitochondria and to insufficient ATP delivery to presynaptic terminals, among the most energy-intensive compartments [40,211]. Under these conditions, presynaptic boutons can experience local ATP scarcity during high-frequency firing, destabilizing vesicle cycling and short-term plasticity. Bioenergetic failure is directly translated into abnormal excitability via ATP-sensitive K^+^ (K_ATP_) channels and ion pumps. Normally, K_ATP_ channels couple intracellular ATP levels to membrane excitability, while Na^+^/K^+^-ATPase maintains resting potential and ionic gradients. Chronic mitochondrial dysfunction destabilizes both systems: maladaptive K_ATP_ activity and reduced Na^+^/K^+^-ATPase function promote Na^+^ and Ca^2+^ accumulation, perturb membrane potential, and favor aberrant burst firing, pushing local circuits toward hyperexcitability [212,213].

Fast-spiking parvalbumin-positive interneurons are especially sensitive to this energetic stress: their high firing rates and dense axonal arborizations make them exceptionally metabolically demanding, and their dysfunction disrupts E/I balance and gamma oscillations, leading to pathological hypersynchrony in vulnerable hubs such as the hippocampus and perirhinal cortex [214,215,216]. This interneuron-centered E/I imbalance mechanism is therefore not an alternative to mitochondrial models but rather a key route through which mitochondrial energetic constraints manifest as circuit-level hyperexcitability.

Over time, repeated episodes of glutamate-driven hyperexcitability on this fragile metabolic background deplete reserves, lower the threshold for excitotoxic cascades, and erode network hierarchies [217]. Ultimately, mitochondrial fragility transforms astrocyte-driven glutamate dysregulation into a neuron-centered failure of ion homeostasis and firing control: inputs handled as physiological activity in healthy networks now precipitate excitotoxic Ca^2+^ overload and bioenergetic collapse, culminating in a progressive “circuit silencing” that underlies severe cognitive decline.

### 3.2. Mitochondria as Gatekeepers of Glutamate-Driven Hyperexcitability and Excitotoxicity

As detailed in Section 2, mitochondrial Ca^2+^ handling defines the physiological window within which glutamatergic activity can be metabolically supported. Under pathological conditions—such as sustained glutamate spillover, uncontrolled glial release, and/or impaired clearance—this buffering window can collapse, and mitochondrial responses increasingly influence whether circuits remain in a hyperexcitable yet reversible state or transition into excitotoxicity. Excessive activation of ionotropic glutamate receptors drives persistent Na^+^ and Ca^2+^ influx and sharply increases metabolic demand. When Ca^2+^ rises beyond the range that mitochondria can safely buffer, the same signaling pathways that normally stabilize synaptic function are thought to become self-amplifying, linking cytosolic Ca^2+^ overload to redox imbalance, energetic failure, and the activation of cell-death cascades [172,218,219,220]. Conceptually, we frame this process as a continuum with a threshold-like transition rather than as two strictly separable states. For clarity, however, we discuss two regimes that capture the dominant system behavior on either side of this threshold: (i) a hyperexcitability regime, in which mitochondrial responses remain adaptive; and (ii) an excitotoxic regime, in which the same responses become self-amplifying and destructive.

#### 3.2.1. Hyperexcitability Regime: Adaptive Ca^2+^-Metabolic Coupling

In the hyperexcitability regime, mitochondria still align ATP production and Ca^2+^ clearance with increased glutamatergic load. Glutamate-induced cytosolic Ca^2+^ transients, entering via NMDARs and VGCCs, are taken up through the MCU complex and stimulate IMM carriers and TCA cycle dehydrogenases, boosting OXPHOS in proportion to synaptic demand [172,221,222]. MCU regulators such as MICU1/2 and MICU3 further tune this coupling, setting the Ca^2+^ threshold at which ATP production is upregulated [172]. These activity-to-metabolism coupling principles are well supported, although the quantitative contribution of individual MCU regulators is likely to vary across cell types and compartments.

In parallel, glutamate can act as an anaplerotic substrate: when pyruvate utilization is limited, glutamate oxidation in the TCA cycle may temporarily sustain ATP production while depleting cytosolic glutamate available for vesicle loading, thereby dampening excitotoxic drive [223,224]. This strategy depends on Ca^2+^-regulated enzymes (e.g., oxoglutarate dehydrogenase) and on the glutamate–aspartate carrier Aralar, activated by Ca^2+^ in the intermembrane space [55,225]. In mitochondrial pyruvate carrier (MPC1) knockout models, or under hypoxia, glutamate oxidation is curtailed, glutamate homeostasis is profoundly altered, and metabolic flexibility is lost [223,224,226,227,228].

When this Ca^2+^-metabolic coupling is compromised—because mitochondrial defects reduce the driving force for Ca^2+^ entry, limit MCU activity, or fragment the network—activity-dependent Ca^2+^ transients no longer elicit sufficient ATP synthesis. Ca^2+^ clearance slows, Na^+^/K^+^-ATPase function deteriorates, and neurons enter an energetically fragile state in which even physiologically plausible activity patterns approach the excitotoxic threshold [229,230,231,232,233]. The increased sensitivity of cortical neurons from β-glucocerebrosidase 1 (GBA1)-null or presenilin-2 (PS2) mutant mice to low micromolar—or even submicromolar—glutamate, despite near-normal ATP/ADP ratios at rest, and the rescue by restoration of pyruvate flux, illustrate how mitochondrial defects can remain silent under baseline conditions, yet profoundly lower the safety margin for glutamatergic signaling [232,233,234].

#### 3.2.2. Excitotoxic Regime: Ca^2+^ Overload, ROS, and Mitochondrial Collapse

In the excitotoxic regime, mitochondria become both targets and amplifiers of glutamate toxicity. Excessive Ca^2+^ influx—particularly via eNMDARs—activates catabolic enzymes (proteases, lipases, and nucleases) and Ca^2+^-dependent phospholipases [PLA_2_ (phospholipase A_2_), COX-2 (Cyclooxygenase-2)], and lipoxygenases that generate eicosanoids and robust ROS production [235]. Within this cascade, prostaglandin E_2_ (PGE_2_) acts as a dose-dependent switch: nanomolar concentrations can be neuroprotective, whereas the micromolar levels reached in excitotoxic conditions promote neuronal death [236].

Within postsynaptic NMDAR–PSD-95 (postsynaptic density protein 95) nanodomains, neuronal nitric oxide synthase (nNOS) senses high local Ca^2+^ and produces nitric oxide (NO), which inhibits complex IV, reacts with superoxide to form peroxynitrite, and can further enhance glutamate release [237]. In parallel, NMDAR-driven Ca^2+^ influx activates a phosphoinositide 3-kinase (PI3K)–PKC pathway that assembles NADPH oxidase (NOX), providing an additional, extramitochondrial source of superoxide; PI3K inhibition prevents NOX activation and cell death without affecting the initial Ca^2+^ rise or mitochondrial depolarization [238,239]. Oxidative DNA damage can then engage PARP-1, which consumes cytosolic NAD^+^ to synthesize poly(ADP-ribose) (PAR) chains. PARP-1 hyperactivation depletes NAD^+^, cripples glycolysis, and deprives mitochondria of pyruvate, thereby promoting mitochondrial depolarization and energy collapse [240].

At the level of Ca^2+^ handling, mitochondria initially take up cytosolic Ca^2+^ via MCU, but matrix Ca^2+^ elevation is itself a potent trigger of mPTP opening, which collapses ΔΨm, halts OXPHOS, and can promote the release of pro-apoptotic factors, with reverse-mode ATP synthase activity accelerating ATP hydrolysis (reviewed in [172,218,219,220]). Genetic manipulation of MCU and NCLX illustrates the double-edged nature of mitochondrial Ca^2+^ flux: reducing MCU expression limits Ca^2+^ loading and protects against acute excitotoxic stimuli [62], whereas MCU overexpression promotes neuronal loss and gliosis [83]. Conversely, constitutive MCU knockout or developmental modulation of NCLX induces profound metabolic adaptations and context-dependent outcomes [241,242] (see also Section 4). Members of the Bcl-2 family add another layer of regulation to mitochondrial Ca^2+^ fluxes, and Bax-deficient mice show protection from delayed Ca^2+^ deregulation after NMDA exposure [243].

Overall, both insufficient and excessive mitochondrial Ca^2+^ uptake—failure to fuel metabolism on one side and Ca^2+^ overload on the other—represent two faces of the same excitotoxic process. The consequence of a given glutamate stimulus critically depends on the pre-existing mitochondrial state: neurons with impaired mitochondrial metabolism or reduced respiratory reserve reach excitotoxic thresholds at much lower, even near-physiological, glutamate levels [229,230,231,232,233]. At the circuit level, maladaptive plasticity and loss of inhibitory restraint can propagate these local perturbations, generating network hyperexcitability that further amplifies glutamate release and metabolic stress. In summary, excitotoxicity could reflect a mismatch between glutamatergic demand and mitochondrial capacity, rather than absolute glutamate levels per se.

### 3.3. Case Study: Alzheimer’s Disease

Dementia affects an estimated 50 million people worldwide, with AD accounting for ~60–80% of cases. Classically, AD is defined by extracellular Aβ plaques and intracellular neurofibrillary tangles composed of hyperphosphorylated Tau. A small fraction of patients carries autosomal-dominant mutations in APP (amyloid precursor protein), PSEN1 (presenilin 1), or PSEN2 (presenilin 2) that cause familial AD (FAD; ~2% of cases), whereas the vast majority present with late-onset, sporadic AD (SAD), in which risk reflects age, environmental factors, and susceptibility alleles. Among these, APOE stands out: APOE ε4 is the strongest common genetic risk factor, APOE ε2 is relatively protective, and APOE ε3 is the most frequent isoform [244,245].

Despite decades of work on amyloid and Tau, no current therapy halts or reverses disease progression, prompting a shift toward earlier, dynamic mechanisms [246,247]. Converging clinical and experimental evidence increasingly positions AD as a glutamate-driven synaptopathy in which mitochondrial dysfunction, hyperexcitability, and excitotoxicity can interact over years. Synaptic loss, rather than overt neuronal death, is the earliest and strongest correlate of cognitive decline, and Aβ/Tau are increasingly viewed as upstream triggers of the triad described above [41,247].

#### 3.3.1. Early Hyperexcitability and Glutamatergic Stress

Clinical and experimental studies reveal that neuronal and network hyperexcitability emerge early in both FAD and SAD. Patients with mild cognitive impairment or presymptomatic mutation carriers often show hippocampal and parahippocampal hyperactivation, while epidemiological and EEG studies document an increased prevalence of subclinical epileptiform discharges and seizures, particularly in early-onset and *PSEN1/2*-linked FAD. Parallel findings in APP/PS1, PS2, Tau, and APOE ε4 mouse models—spontaneous non-convulsive seizures, cortical and hippocampal hypersynchrony, and clusters of hyperactive neurons near plaques—together with hyperexcitable phenotypes in induced pluripotent stem cell (iPSC)-derived neurons and organoids carrying FAD mutations or APOE ε4 indicate that excitability changes are at least partly cell-intrinsic [42,248,249].

Within this framework, a “glutamatergic hypothesis” of AD has emerged. AD is viewed, at least in part, as a state of chronic glutamate-dependent hyperexcitability that gradually crosses the threshold into excitotoxicity [42]. Early in the disease, mitochondrial and astroglial safeguards can still maintain ion homeostasis and keep hyperexcitability reversible. Over time, however, Aβ, Tau, presenilin mutations, and APOE ε4 progressively erode glutamate clearance mechanisms and weaken mitochondrial resilience, thereby lowering the excitotoxic threshold [42,248,249].

#### 3.3.2. Mitochondrial Contributions to Vulnerability

Mitochondrial dysfunction is an early and persistent feature of AD. The classical “Ca^2+^ cascade” and “mitochondrial cascade” hypotheses proposed that neurons in aged and AD brains develop exaggerated cytosolic Ca^2+^ elevations upon depolarization, and that excessive mitochondrial Ca^2+^ uptake then drives ROS production, ATP depletion, mPTP opening, and caspase activation [55]. More recent work refines this view: both mitochondrial Ca^2+^ overload and blunted mitochondrial Ca^2+^ uptake have been described, and either extreme can promote neuronal hyperexcitability and vulnerability to glutamate excitotoxicity [172].

Aβ oligomers can translocate to mitochondria, interact with cyclophilin D, favor mPTP opening, and promote matrix Ca^2+^ overload, ΔΨm collapse, and cytochrome *c* release [187,234,250,251,252,253,254]. In parallel, *PSEN2* mutations and MPC1 dysfunction reduce mitochondrial pyruvate uptake and blunt activity-dependent mitochondrial Ca^2+^ signals: ATP levels remain near-normal at rest but collapse rapidly under even moderate glutamatergic challenges, and restoring mitochondrial pyruvate flux rescues these phenotypes [229,230,231,232,233]. Conversely, some models show elevated basal mitochondrial Ca^2+^ and MCU upregulation after plaque deposition, with genetic or pharmacological MCU inhibition normalizing AD-associated hallmarks [250,251,252,255,256].

Despite mechanistic heterogeneity, the functional outcome converges: synaptic mitochondria fail to match metabolic output to glutamatergic demand, sensitizing synapses to Ca^2+^-dependent degeneration. Presynaptically, modest Ca^2+^ elevations drive excessive or asynchronous glutamate release; postsynaptically, slower Ca^2+^ decay promotes recruitment of toxic cascades (PLA_2_/COX-2/lipoxygenases, nNOS/NO, NADPH oxidase activation, PARP-1–dependent NAD^+^ depletion), producing repeated bouts of partial mitochondrial depolarization rather than a single catastrophic insult.

Rather than one terminal event, neurons undergo cycles of incomplete recovery that gradually erode synaptic resilience [218]. Within this framework, synaptic mitochondria set the threshold at which Aβ/Tau-driven hyperexcitability transitions into local excitotoxic synapse loss.

#### 3.3.3. Astrocytic Failure as a Parallel Amplifier

Astrocytes undergo early, progressive dysfunction in AD. EAAT1/EAAT2 expression and glutamate transport decline, PAPs retract, and glutamine synthetase is reduced. These alterations expand the extracellular glutamate pool, weaken inhibitory restraint, and facilitate eNMDAR recruitment [257,258,259,260,261,262]. Aβ oligomer accumulation in astrocytes can impair the ETC, reduce ATP availability, and compromise the energetically expensive operation of EAAT2/GLT-1, while Aβ-activated microglia release TNF-α, which promotes ubiquitin-dependent degradation of EAAT2 [263,264]. As PAPs retract and EAAT2 is lost, glutamate spillover activates neighboring synapses and eNMDARs, increases ambient glutamate, and injects “synaptic noise” into circuits [204,205]. Reactive astrogliosis can further impair K^+^ buffering (e.g., via Kir4.1 downregulation) and reduce glutamine supply to inhibitory interneurons, leading to smaller inhibitory postsynaptic currents and network hyperexcitability that can be rescued by glutamine supplementation [265,266,267].

Taken together, clinical, experimental, and human cell-based data support a coherent picture: early in the AD trajectory, glutamate-driven hyperexcitability is a robust phenotype generated by coordinated changes at presynaptic terminals, postsynaptic receptors, and astrocytic clearance pathways. These mechanisms converge on mitochondrial Ca^2+^ handling and bioenergetic support at the tripartite synapse as a shared vulnerability node.

#### 3.3.4. A Mitochondria-Centered Synaptopathy

Conceptually, these observations support the idea that the classical “Ca^2+^ cascade” and “mitochondrial cascade” hypotheses converge on a glutamate-driven, mitochondria-centered synaptopathy rather than a simple, one-directional Ca^2+^ overload model. MERCs emerge as a potential structural hub where Aβ, presenilins, and Tau may alter Ca^2+^ transfer, while synaptic mitochondria help determine whether persistent hyperexcitability remains reversible or evolves into chronic, subthreshold excitotoxicity [172,268,269].

Early in the disease trajectory—mild cognitive impairment, presymptomatic carriers, young APOE ε4 individuals—networks show hyperactivity and hypersynchrony, which boosts Aβ production, promotes trans-synaptic Tau spread and repeatedly loads synaptic mitochondria with Ca^2+^ and ROS [270,271,272,273,274]. Over time, recurrent mitochondrial stress depletes bioenergetic reserves, lowers excitotoxic thresholds, and drives selective synapse loss, culminating in circuit “silencing” characteristic of late-stage AD [42,275]. In this view, mitochondria at the glutamatergic tripartite synapse function as critical modulators—and, under sustained stress, executors—of glutamate toxicity. Stabilizing excitability in AD will likely require co-targeting glutamate receptors and transporters together with mitochondrial Ca^2+^ handling and astrocytic metabolism, rather than focusing on neuronal receptors in isolation. Collectively, these processes may shift astrocytes from stabilizers to amplifiers of glutamatergic stress, lowering the threshold for synaptic and metabolic failure in AD.

## 4. Translational Outlook

From a translational perspective, this framework offers a unifying lens to interpret how diverse brain disorders emerge from shared vulnerabilities in mitochondrial Ca^2+^ handling, redox balance, and metabolic flexibility.

AD provides a paradigmatic example. Across clinical and preclinical experimental studies, glutamate-driven hyperexcitability emerges as an early and robust feature, and canonical amyloid- and Tau-related pathways, together with genetic risk factors, converge on mitochondrial stress at the tripartite synapse [41,42,248,249]. A similar logic appears to apply to epilepsy, amyotrophic lateral sclerosis (ALS), and ischemia, where impaired astrocytic glutamate clearance, interneuron dysfunction, and mitochondrial fragility convert physiological glutamatergic signaling into progressive excitotoxic synaptopathy [78,192,202,219].

Across disorders, neuronal and astrocytic mitochondria normally enable circuits to tolerate substantial glutamatergic load and return to baseline once activity subsides. However, when Ca^2+^ handling, redox balance, or metabolic flexibility are compromised, the same glutamatergic drive is more likely to push the system across the excitotoxic threshold, leading to ATP failure, ROS amplification, mPTP opening, and delayed cell death [201,202,276].

Despite this conceptual convergence, important gaps remain. Many mechanistic insights derive from in vitro or ex vivo preparations exposed to non-physiological stimulation and rarely incorporate simultaneous monitoring of cytosolic and mitochondrial Ca^2+^, metabolism, and ROS in identified cell types. In particular, we lack quantitative, cell type- and compartment-resolved definitions of “physiological” versus “pathological” mitochondrial Ca^2+^ and ROS signaling, including how these signals are distributed across presynaptic boutons, postsynaptic spines, fine astrocytic processes, and perivascular endfeet. Similarly, the relative contributions of aspartate aminotransferase (AAT) versus GDH flux, the role of pyruvate recycling during physiological activity, and the precise topology linking glucose oxidation to glutamate–glutamine cycling remain largely inferred rather than directly measured in vivo.

These conceptual gaps are compounded by technical constraints. Existing Ca^2+^ and redox reporters are limited in dynamic range, pH sensitivity, and specificity for mitochondrial versus NOX-derived ROS, and most pharmacological tools (e.g., MCU/NCLX modulators, antioxidants, metabolic drugs) lack brain or cell-type specificity.

Addressing these challenges will require experimental designs that explicitly respect the spatial and cell-type compartmentalization of the mitochondrial–glutamatergic axis. Priority directions include fast, multiplexed genetically encoded sensors for Ca^2+^, NADH/NAD^+^, ATP, and ROS targeted to mitochondrial, cytosolic, synaptic, and perivascular domains; in vivo and ex vivo imaging strategies that combine these reporters with two-photon and mesoscale readouts of glutamatergic activity and network dynamics; and cell- and compartment-specific manipulation of Ca^2+^ transporters, MERC tethers, and metabolic enzymes using genetic tools.

Longitudinal studies in genetic and sporadic models will be crucial to map when and where mitochondrial Ca^2+^ and redox signaling first become dysregulated along the hyperexcitability–excitotoxicity trajectory.

Consistent with this view, translational strategies should prioritize stabilization of the mitochondrial–glutamatergic axis rather than targeting glutamate receptors or transporters in isolation. Current approaches converge on several promising directions: (i) modulating mitochondrial dynamics and quality control (for example, limiting excessive fission and enhancing mitophagy); (ii) reinforcing bioenergetic and redox capacity through mitochondrial biogenesis or metabolic interventions such as ketogenic or anaplerotic support; and (iii) fine-tuning mitochondrial Ca^2+^ handling through MCU-complex regulators. More recently, astrocyte-focused gene therapies using adeno-associated virus (AAV) vectors and cell-specific promoters, as well as experimental strategies for mitochondrial transfer or transplantation, have highlighted the feasibility of directly targeting mitochondrial function at the astrocyte–neuron interface. These approaches show encouraging preclinical efficacy in stabilizing energy metabolism, preserving glutamate transporter expression, and raising the threshold for excitotoxic injury in models of epilepsy, ischemia, and neurodegeneration [277,278,279].

Importantly, the same experimental tools that now allow real-time visualization of mitochondrial Ca^2+^, ATP, and redox state at synaptic resolution may ultimately yield mitochondria-informed biomarkers to stratify patients by “mitochondrial reserve” and monitor target engagement in early-phase trials. A key opportunity will be to link subcellular mitochondrial readouts to circuit-level phenotypes—such as hyperexcitability, impaired plasticity, and altered neurometabolic coupling—so that mitochondrial interventions can be evaluated not only by pathological endpoints, but also by their ability to restore physiological operating ranges of activity and energy use.

Overall, across glutamate-driven brain disorders, mitochondrial capacity to buffer Ca^2+^, sustain metabolism, and control redox signaling emerges as a central determinant of whether hyperexcitability remains reversible or progresses toward excitotoxic degeneration, identifying the mitochondrial–glutamatergic axis as a unifying and actionable translational target.

## 5. Conclusions

Mitochondria are not only cellular powerhouses but also pivotal metabolic and redox integrators. Their ROS output—tuned by Ca^2+^ uptake, respiratory flux, and local antioxidant capacity—acts as a short-range signal that adjusts receptor function, synaptic strength, and metabolic output across the tripartite synapse. In neurons, mitochondrial ROS couple glutamate receptor activation and Ca^2+^ entry to AMPAR/NMDAR gating, trafficking, and plasticity, whereas in astrocytes, a looser respiratory organization and tonic ROS output support Nrf2-dependent antioxidant programs, glutathione shuttling, and neurometabolic coupling. When this balanced “oxidative eustress” is exceeded—by Ca^2+^ overload, impaired glutamate clearance, chronic inflammation, or disease-linked proteins—these signals can shift toward oxidative distress, amplifying glutamatergic dysregulation, excitotoxicity, and vulnerability to neurodegeneration.

Within this conceptual framework, insufficient or excessive mitochondrial Ca^2+^ uptake, as well as physiological and pathological ROS, reflect points along a continuum rather than discrete or mutually exclusive states. Accordingly, hyperexcitability and excitotoxicity represent distinct operational states, defined by the capacity of astrocytes and mitochondria to sustain glutamatergic load and restore homeostasis following activity.

Taken together, these observations support the view that mitochondria act as gatekeepers that determine whether chronic hyperexcitability remains reversible or evolves into local synapse loss and network “silencing”.

More broadly, mitochondria stand at the intersection of metabolism, signaling, and plasticity—not merely as downstream victims but as bona fide biosensors and regulators of synaptic health. A major challenge for the next decade will be to move the glutamate–mitochondria axis from a largely descriptive framework to a quantitatively grounded and therapeutically actionable target. This will require defining what “physiological” mitochondrial signaling looks like in each compartment, identifying the earliest points of failure along the hyperexcitability–excitotoxicity trajectory, and determining which components can be safely tuned without destabilizing network function. Achieving this goal will require integration across subcellular, cellular, and network scales and the development of interventions that restore global mitochondrial homeostasis across neurons, astrocytes, and microglia, rather than correcting isolated nodes. If successful, such strategies may transform the mitochondrial network from a locus of vulnerability into a cornerstone of therapeutic resilience for glutamate-driven brain disorders.

## Figures and Tables

**Table 1:** Compartment-specific mitochondrial contributions to glutamate handling and ionic homeostasis at the tripartite synapse.

Compartment	Pathway/Process	Mitochondria-Linked Function
Presynaptic neuron	Transmitter supply (glutamate cycle)	Mitochondria-associated glutaminase (PAG/GLS) converts glutamine to glutamate, supporting the presynaptic transmitter pool for vesicle loading.
	Vesicle cycle and release	ATP supports V-ATPase–driven vesicle reacidification, loading, docking/priming, endocytosis and recycling. MCU–NCLX Ca^2+^ cycling buffers local Ca^2+^ signals, stabilizes release probability and limits asynchronous release during repetitive firing.
	Ionic homeostasis	ATP sustains Na^+^/K^+^-ATPase activity to restore presynaptic ionic gradients during sustained activity.
Postsynaptic neuron	Signal decoding and plasticity	Postsynaptic mitochondria buffer NMDAR/VGCC Ca^2+^ microdomains, shaping Ca^2+^ spread and decay and preserving biochemical compartmentalization across spines and dendrites.
	Energy for synaptic function	ATP supports local ion pumping (Na^+^/K^+^-ATPase, PMCA), receptor trafficking, actin remodeling and activity-dependent protein synthesis required for LTP/LTD stabilization.
	Glutamate metabolism (matrix catabolism)	Mitochondria convert glutamate to α-ketoglutarate (α-KG) via aminotransferases (e.g., GOT2/AAT2) and/or glutamate dehydrogenase (GDH), feeding the TCA cycle and linking activity to metabolic flux.
Astrocyte (PAPs)	Glutamate uptake (clearance)	EAAT1/GLAST and EAAT2/GLT-1 clear glutamate via electrogenic co-transport (3 Na^+^ + 1 H^+^ in/1 K^+^ out per glutamate), imposing a large Na^+^ load that requires Na^+^/K^+^-ATPase. Mitochondrial ATP (directly and/or via mitochondria-supported glycolysis) sustains the energetic demand.
	Recycling (glutamate–glutamine cycle)	Glutamate is converted to glutamine via GS (ATP-consuming). Glutamine is returned via SNATs (e.g., SNAT3 in astrocytes; SNAT1/2 in neurons), sustaining transmitter replenishment.
	Metabolic coupling	Astrocytic glycolysis/glycogenolysis provides rapid ATP. Mitochondria contribute oxidative capacity and influence pyruvate oxidation versus lactate production. Lactate export via MCT1/4 supports neuronal oxidative metabolism (context-dependent ANLS).
	Ca^2+^ microdomains and gliotransmission	Mitochondrial Ca^2+^ buffering and redox tone shape perisynaptic Ca^2+^ microdomains that regulate gliotransmitter release and the stability of uptake/homeostatic programs.
Shared (all compartments)	Ca^2+^ and redox control	MCU–NCLX cycling links Ca^2+^ signals to ATP production. Controlled ROS supports signaling (“oxidative eustress”).
	*Ionic gradient restoration*	ATP supply supports major ion pumps/exchangers (Na^+^/K^+^-ATPase, PMCA; SERCA), preserving excitability set-points.

**Table 2:** Mitochondria-dependent mechanisms linking glutamate homeostasis disruption to hyperexcitability and excitotoxicity in neurons and astrocytes. Symbols indicate directionality of change: ↑ increased; ↓ decreased.

Compartment	Primary Mitochondrial Failure Features	Consequences for Glutamate/Ion Homeostasis	Hyperexcitability/Excitotoxic Outcome
Presynaptic neuron	↓ ATP; impaired MCU–NCLX Ca^2+^ cycling; ↑ ROS; ΔΨm loss; ↑ mPTP propensity	Impaired vesicle reacidification/refilling, endocytosis and reserve-pool mobilization. Presynaptic Ca^2+^ accumulation and reduced buffering.	Increased spontaneous/asynchronous release. Unstable high-frequency transmission. Stress-triggered increases in glutamate output that accelerate network hyperexcitability.
Postsynaptic neuron	↓ ATP; prolonged Ca^2+^ transients (reduced buffering/efflux); ↑ ROS; ΔΨm loss; mPTP opening	Reduced ion-pump capacity (Na^+^/K^+^-ATPase/PMCA) and impaired membrane potential restoration. Sustained Ca^2+^ elevation promotes toxic signaling cascades.	Recruitment of pro-death pathways downstream of intense NMDAR/eNMDAR activation (e.g., nNOS/NO, NOX, lipid peroxidation, PARP-1). Spine instability and synapse loss.
Astrocyte (PAPs)	↓ ATP; Oxidative stress; Altered mitochondrial Ca^2+^ handling; ΔΨm loss; mPTP flickering dysregulation	Reduced EAAT-mediated clearance and Na^+^/K^+^-ATPase support. Impaired K^+^ buffering (often coupled to reactive changes); Reduced GS activity and altered glutamine supply.	Elevated ambient glutamate and spillover to eNMDAR recruitment. Reactive gliotransmission (Cx43 hemichannels, pannexin-1, Best1) releasing glutamate/ATP/D-serine. Amplification of network synchrony and excitotoxic drive.
Common/circuit-level effects	System-wide energetic failure; ROS amplification; Ca^2+^ dysregulation.	↑ extracellular glutamate (spillover, impaired uptake). ↑ intracellular Na^+^/Ca^2+^. Collapse of ionic gradients. Impaired inhibition (E/I imbalance).	Lowered excitotoxic threshold: hyperexcitability becomes self-reinforcing. Ca^2+^ overload. mPTP opening. Cell death. Oxidative damage to proteins/lipids/DNA. Progressive circuit “silencing”.

## Data Availability

No new data were created or analyzed in this study.
